# Supplementary material for: Establishment of an Absolute Quantitative Method to Detect a Plasma tRNA-Derived Fragment and Its Application in the Non-Invasive Diagnosis of Gastric Cancer
Source: Int J Mol Sci. 2022 Dec 24;24(1):322. doi: 10.3390/ijms24010322 (PMC9820402; doi:10.3390/ijms24010322)
Supplement: Supplementary file 1 [file ijms-24-00322-s001.zip › ijms-2033529-supplementary.pdf]

Supplementary Material

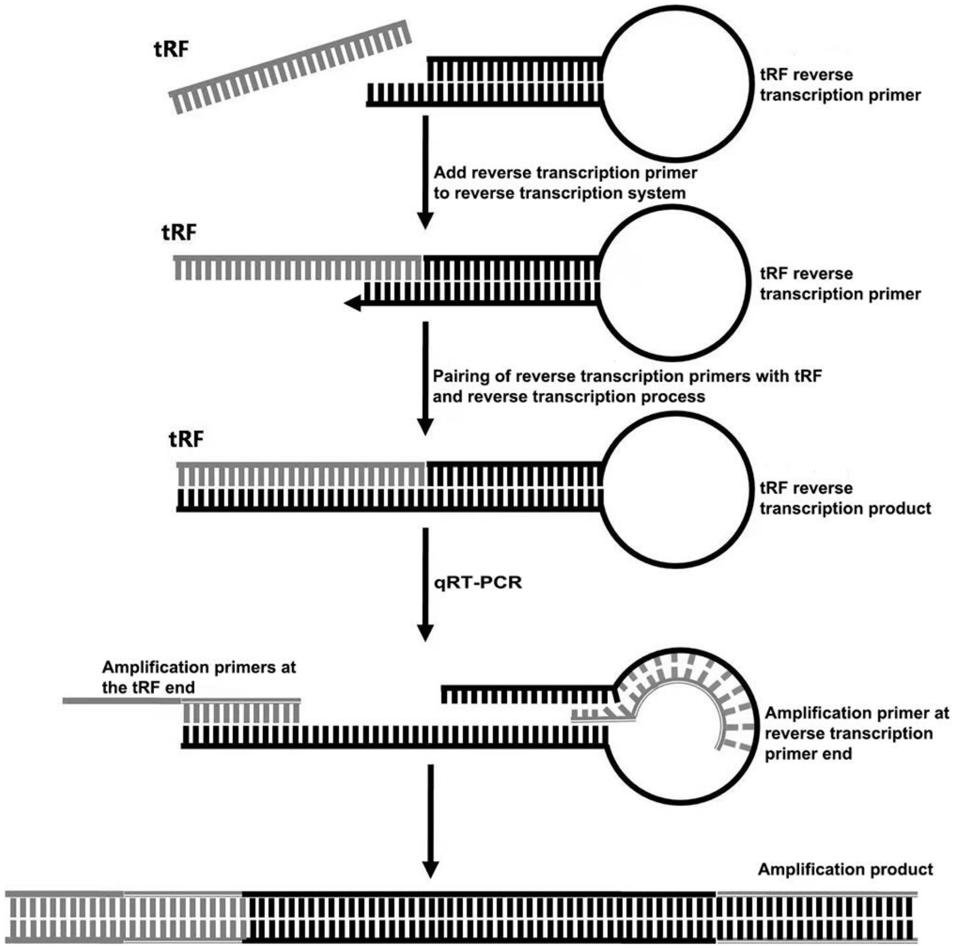

Supplementary Figure S1. Principle of designing reverse transcription and amplify primers based on stem-loop structure.

Supplementary Table S1. Reverse transcription reaction system

Component	Volume (μL)
Total RNA	6
5×Polestar RT MasterMix (with dsDNase)	4
Stem-loop structure reverse transcription primer for tRF-27 (0.005μM)	2
RNase Free H_2O	8
Total volume	Up to 20

Supplementary Table S2. Reverse transcription program

Step	Temperature (°C)	Time (min)
cDNA synthesis	37	30
Deactivated transcriptase	85	5

Supplementary Table S3. TaqMan probe reaction system

Component	Volume (μL)
2×5G qPCR PreMix (QPT-200, Toyobo, Japan)	10
Forward primer (10 μM)	1.4
Reverse primer (10 μM)	1.4
TaqMan Probe (10 μM)	0.8
cDNA template	0.8
ddH_2O	5.6
Total volume	20

Supplementary Table S4. TaqMan probe reaction program

Step	Temperature (°C)	Time (Sec)	Cycles
Pre-denaturation	95	30	1
Denaturation	95	10	40
Extension/annealing	60	20	40
